# Wnt3a Protein Reduces Growth Factor-Driven Expansion of Human Hematopoietic Stem and Progenitor Cells in Serum-Free Cultures

**DOI:** 10.1371/journal.pone.0119086

**Published:** 2015-03-25

**Authors:** Lucia E. Duinhouwer, Nesrin Tüysüz, Elwin W. J. C. Rombouts, Mariette N. D. ter Borg, Enrico Mastrobattista, Jan Spanholtz, Jan J. Cornelissen, Derk ten Berge, Eric Braakman

**Affiliations:** 1 Department of Hematology, Erasmus University Medical Centre, Rotterdam, The Netherlands; 2 Erasmus MC Stem Cell Institute, Erasmus University Medical Centre, Rotterdam, The Netherlands; 3 Institute for Pharmaceutical Sciences, Utrecht University, Utrecht, The Netherlands; 4 Glycostem, Oss, The Netherlands; French Blood Institute, FRANCE

## Abstract

Ex vivo expansion of hematopoietic stem and progenitor cells (HSPC) is a promising approach to improve insufficient engraftment after umbilical cord blood stem cell transplantation (UCB-SCT). Although culturing HSPC with hematopoietic cytokines results in robust proliferation, it is accompanied with extensive differentiation and loss of self-renewal capacity. Wnt signaling has been implicated in regulating HSPC fate decisions in vivo and in promoting HSPC self-renewal by inhibition of differentiation, but the effects of Wnt on the ex vivo expansion of HSPC are controversial. Here, we demonstrate that exogenous Wnt3a protein suppresses rather than promotes the expansion of UCB-derived CD34^+^ cells in serum free expansion cultures. The reduced expansion was also observed in cultures initiated with Lin^-^CD34^+^CD38^low^CD45RA^-^CD90^+^ cells which are highly enriched in HSC and was also observed in response to activation of beta-catenin signaling by GSK3 inhibition. The presence of Wnt3a protein during the culture reduced the frequency of multilineage CFU-GEMM and the long-term repopulation ability of the expanded HSPC. These data suggest that Wnt signaling reduces expansion of human HSPC in growth factor-driven expansion cultures by promoting differentiation of HSPC.

## Introduction

Allogeneic hematopoietic stem cell transplantation is an important part of treatment for patients suffering from hematological disorders, including leukemia, myelodysplastic syndromes, and aplastic anemia. However, many patients lack a suitable sibling or human leucocyte antigen (HLA) matched unrelated donor. Because of its rapid availability and less stringent matching criteria[1], umbilical cord blood (UCB) is an important alternative source for hematopoietic stem and progenitor cells (HSPC). However, UCB-derived HSPC significantly differ from bone marrow- and peripheral blood-derived HSPC quantitatively and qualitatively. UCB grafts contain a relatively low number of HSPC which are relatively more primitive, resulting in impaired engraftment and a delayed hematopoietic recovery[1–5], during which patients are at increased risk for severe complications, including infections and bleeding. Several approaches have been pursued to improve engraftment after UCB transplantation, including the *ex vivo* expansion of HSPC.

HSC are defined by their self-renewal capacity and the ability to generate all different hematopoietic lineages. Although *in vivo* studies demonstrated that HSPC expand after transplantation[6], robust *ex vivo* expansion of long-term repopulating HSC remains a challenge. Culturing HSPC with different combinations of hematopoietic cytokines such as stem cell factor (SCF), Fms-related tyrosine kinase 3 ligand (Flt3L), thrombopoietin (TPO) and granulocyte-macrophage colony-stimulating factor (GM—CSF) resulted in massive expansion of committed HPC which is accompanied by a loss or at best maintenance of primitive HSC with long-term repopulation ability.[7–11]. Additional signals are needed to support the expansion of primitive HSC in *ex vivo* culture systems. Several novel factors, such as the immobilized Notch-ligand Delta1, copper chelator tetra-ethylenepentamine (TEPA) and signals derived from mesenchymal stromal cells, were identified that may affect self-renewal of HSC and inhibit differentiation, thereby having the potential to improve *ex vivo* expansion protocols[12–14]. In addition, numerous promising factors have been tested in a pre-clinical setting, including developmental regulators such as fibroblast growth factor signaling, insulin-like growth factor, Angiopoietin-like proteins and Pleiotrophin and chemical modulators like all-trans retinoic acid, stemregenin1 and prostaglandin E2 (reviewed by Walasek et al.[15]).

The Wnt/beta-catenin signaling pathway regulates cell fate decisions in many developmental processes in embryo and adult. Stimulation of cells with Wnt signaling proteins induces the stabilization and accumulation of the signal transducer protein beta-catenin, which then localizes into the nucleus where it regulates target gene expression (reviewed by Clevers et al.[16]). When combined with other growth factors, Wnt proteins can promote self-renewal in several types of stem cells, such as mammary, intestinal and embryonic stem cells[17–20]. Several studies, using different approaches to inhibit the Wnt signaling pathway, showed that Wnt signaling is pivotal for normal HSC function in mouse[21–23]. In addition, some reports show that treatment with recombinant Wnt3a protein or overexpression of activated beta-catenin enhances the self-renewal capacity of mouse HSC ex vivo[24–26]. These studies offer hope that Wnt signals may be of use in the expansion of human UCB-derived HSPC. However, other studies show that constitutive activation of beta-catenin blocks multilineage differentiation[27] and that active beta-catenin induces apoptosis in HSPC[28, 29].

In this study we investigate the effect of Wnt signals on growth factor-driven ex vivo expansion of human HSPC. We show that Wnt3a signaling reduces growth factor driven expansion of human HSPC by promoting differentiation.

## Material and Methods

### Cord blood processing, CD34+ cell selection and HSC sorting

Umbilical cord blood was collected in several hospitals using Stemcare/CB collect blood bag system (Fresenius Kabi Norge AS) containing citrate phosphate dextrose (CPD) as an anticoagulant. Approval for collection was obtained from the Medical Ethical Committee of the Erasmus University Medical Centre (MEC-2009–410) and written informed consent from the mother was obtained prior to donation of the cord blood. Within 48 hours after collection, mononuclear cells were isolated using ficoll (Lymphoprep, Fresenius Kabi Norge AS). CD34^+^ cells were isolated with double positive immunomagnetic selection using Magnetic Activated Cell Sorting (MACS) technology according instructions of the manufacturer (Miltenyi Biotech GmBH, Bergisch Gladbach, Germany). MACS-selected CD34^+^ cells were either used directly in experiments or stained with anti-Lin-FITC, anti-CD38-PerCP-Cy5.5, anti-CD90-PE (all from eBioscience, Vienna, Austria), anti-CD34-PE-Cy7, anti-CD45RA-APC-H7 (both from BD Biosciences, San Jose, CA, USA) and DAPI (Sigma-Aldrich, St Louis, MO, USA) after which viable DAPI^-^Lin^-^CD34^+^CD38^low^CD45RA^low^CD90^+^-cells, highly enriched for hematopoietic stem cells (HSC)[30], were sorted using BD FACSAria Cell Sorting System (BD Biosciences, San Jose, CA, USA).

### Expansion cultures

Selected CD34+ cells and sorted DAPI^-^Lin^-^CD34^+^CD38^low^CD45RA^low^CD90^+^-cellswere cultured in serum free Glycostem Basic Growth Medium (GBGM, Glycostem, Oss, The Netherlands) or StemSpan Serum-Free Expansion Medium (SFEM, Stemcell Technologies, Grenoble, France) supplemented with 20 μg/ml low molecular weight heparin (Abbott, Wiesbaden, Germany) and the early acting growth factors SCF (50 ng/ml, Cellgenix, Freiburg, Germany), Flt3L (50 ng/ml, Cellgenix, Freiburg, Germany) and TPO (50 ng/ml, Cellgenix, Freiburg, Germany) (from now on referred to as ‘SFT medium’) with or without the addition of 250 ng/ml purified Wnt3a unless indicated otherwise. Cells were cultured in a volume of 1 ml in 24-well plate at a concentration of 10^5^/ml at 37°C in 5% CO_2_. Every 2 to 3 days, wells were split or half of the medium was refreshed. In some experiments, we used GSK3β inhibitor CH99021 (1 μM, Stemgent, Cambridge, MA, USA) as an alternative activator of the canonical Wnt pathway. Frizzled8CRD (Fr8CRD, which blocks the binding of Wnt3a to its receptor) was produced as described[31] and used at a concentration of 15 μg/ml. Wnt3a was combined in some experiments with the Aryl hydrocarbon Receptor (AhR) antagonist StemRegenin1 (SR1, 1 μM, Cellagen Technology, San Diego, CA, USA).

### Purification of Wnt3a and preparation of liposomal Wnt3a

Wnt3a-conditioned medium was collected from Drosophila S2 cells grown in suspension culture. Wnt3a was further purified using Blue Sepharose affinity and gel filtration chromatography as described[24]. Liposomes containing DMPC (1,2-dimyristoyl-sn-glycero-3-phosphocholine), DMPG (1,2-dimyristoyl-sn-glycero-3-phospho-rac-glycerol) (both Lipoid AG) and Cholesterol (Sigma-Aldrich, St Louis, MO, USA) at a 10:1:10 molar ratio were prepared by extrusion method. Purified Wnt3a was mixed with liposomes at a 1:7.5 ratio to achieve a total concentration of 7–10 μg/ml of Wnt3a. After mixing, the Wnt liposomes were incubated for at least one hour on the roller coaster at 4°C. Next, CHAPS was removed from the Wnt liposomes by dialysis at least three times in PBS 1 hour each, using dialysis membrane with molecular weight cut-off of 10 kDa at 4°C. The Wnt liposomes were stored at 4°C. Activity of purified Wnt3a protein and liposomal Wnt3a was determined in a luciferase reporter assay (see below).

### Luciferase reporter assay

Mouse LSL cells, which express luciferase in response to TCF promoter binding, were routinely cultured at 37°C and 5% CO2 in culture medium composed of DMEM (Invitrogen, Life Technologies, Bleiswijk, The Netherlands), 10% FCS, and 1% Penicillin/Streptomycin. For the activity assays, Wnt3a reagents at a concentration of 250 ng/ml were incubated in culture medium without FCS for various periods of time at 37°C in U-bottom 96-well plates. These media were then transferred to F-bottom 96-well plates containing LSL cells, which were plated the day before at a density of 25,000 cells/well. After overnight incubation with the indicated Wnt reagents, relative luciferase units were measured with Glomax multiplate reader.

### Flowcytometry

At serial time points in culture, absolute numbers of viable CD34^+^ cells were determined by a single platform flowcytometric assay, using anti-CD45-FITC, anti-CD34-PE, DAPI and a calibrated number of Stem-Count Fluorospheres (all from Beckman Coulter, Fullerton, CA, USA). Within the CD34^+^ population, the frequency of DAPI^-^Lin^-^CD34^+^CD38^low^CD45RA^low^CD90^+^-cells was determined using the antibody panel as described above for the sorting of these cells. Absolute numbers of DAPI^-^Lin^-^CD34^+^CD38^low^CD45RA^low^CD90^+^-cells were determined by multiplying the absolute number of CD34^+^ cells obtained in the single platform analysis by the percentage of DAPI^-^Lin^-^CD34^+^CD38^low^CD45RA^low^CD90^+^-cells within the CD34^+^ cell population. Flowcytometric analysis was performed using a BD FACSCanto (BD Biosciences, San Jose, CA, USA) and data was analyzed using FlowJo software (Tree Star Inc, Ashland, OR, USA).

### Transplantation of human hematopoietic cells into NOD.Cg-Prkdcscid Il2rgtm1Wjl/SzJ (NSG) mice

This study was carried out in accordance to the Dutch law on Animal Welfare and Experiments. The protocol was approved by the Committee on the Ethics of Animal Experiments of the Erasmus University Medical Centre Rotterdam, The Netherlands. Intrabone transplantations were performed under isoflurane anesthesia. All animals were housed in groups in individually ventilated cages. Food and water were available ad libitum.

NSG mice were sublethally irradiated (3 Gy) and subsequently transplanted with the progeny generated from 1,00E+05 UCB-derived CD34^+^ cells cultured in our SFT medium, with or without the addition of 250 ng/ml Wnt3a for 7 days. Each group contained 5 mice. Engraftment was assessed every 2 weeks starting at 3 weeks after transplantation by flowcytometric analysis of the peripheral blood, using a flowcytometric panel including anti-mouseCD45-eFluor450, (eBioscience, Vienna, Austria) and anti-humanCD45-APC-Cy7 (BioLegend, London, UK). Mice were considered engrafted when human CD45 levels were higher than 0.1%. At 17 weeks after transplantation, the mice were sacrificed by cervical dislocation and cells from femurs were analysed.

## Results

### Wnt3a reduces growth factor-driven expansion of UCB derived CD34^+^ cells

**Fig 1:**
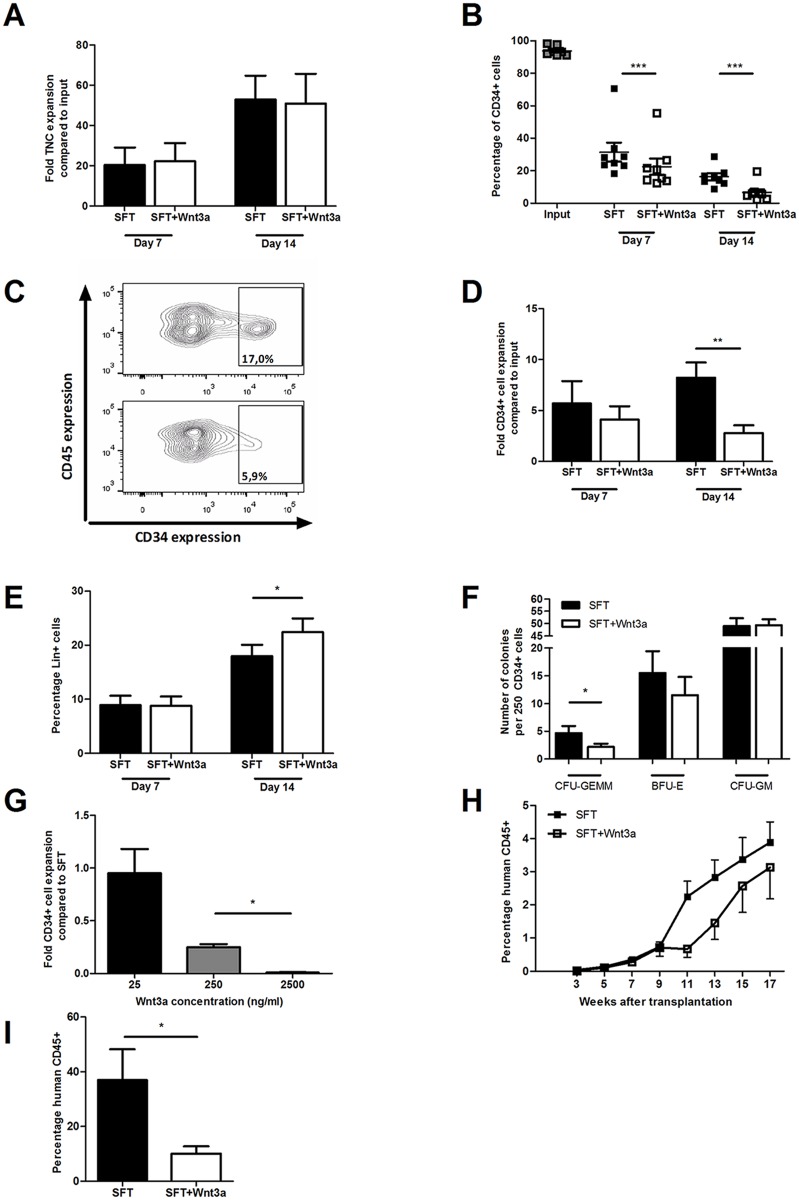
Exogenous Wnt3a reduces growth factor-driven expansion of CD34^+^ cells. UCB-derived CD34^+^ cells were cultured in serum free SFT medium with or without the addition of Wnt3a. Cells were analyzed using flow cytometry at 7 and 14 days of culture. Shown are (A) the total nucleated cell expansion compared to input (n = 8), (B) the frequency of CD34^+^ cells within the TNC population during culture (n = 8), (C) expression of CD45 and CD34 after 14 days of culture in SFT with (lower panel) or without (upper panel) Wnt3a (representative experiment out of 8), (D) the expansion of CD34^+^ cells compared to input (n = 8) and (E) the frequency of cells expressing lineage markers after 7 and 14 days of culture (n = 6). (F) Frequency of CFU-GEMM, BFU-E and CFU-GM in 250 CD34^+^ cells cultured for 2 weeks in SFT or SFT+Wnt3a (n = 2, 3 dishes per experiment). (G) CD34^+^ cell expansion compared to SFT medium after 14 days of culture with different dosages of Wnt3a (n = 2). (H) Levels of human chimerism at several time points after transplantation with the progeny of 10^5^ CD34^+^ cells cultured for 7 days in SFT or SFT+Wnt3a medium (n = 5 mice per group). (I) Levels of human chimerism in bone marrow 17 weeks after transplantation with the progeny of 10^5^ CD34^+^ cells cultured for 7 days in SFT or SFT+Wnt3a medium (n = 5 mice per group). * p<0.*05*, ** p<0.*01*, *** p<0.*001*

To assess whether Wnt signals affect expansion of human HSPC in culture, UCB-derived CD34^+^ cells were cultured in serum-free medium supplemented with SCF, Flt3L and TPO (SFT medium) with or without purified Wnt3a protein. After 14 days, no significant change in total nucleated cell expansion was observed in response to Wnt3a protein (Fig. 1A, p = 0.74). However, Wnt3a accelerated the decline in the frequency of CD34^+^ cells that was observed during culture (Fig. 1B, p<0.001 for both 7 and 14 days of culture and Fig. 1C), resulting in a significantly reduced expansion of CD34^+^ cells after 2 weeks of culture (Fig. 1D). In addition, we observed a higher frequency of cells expressing lineage markers after 14 days of culture in the presence of Wnt3a protein compared with SFT medium only (Fig. 1E, p<0.05). Next, we assessed the functionality of the cultured CD34^+^ cells by performing colony forming unit (CFU) assays. The presence of Wnt3a during culture reduced the frequency of multi-lineage CFU-GEMM (granulocyte, erythrocyte, monocyte, megakaryocyte), while no effect was seen in the frequency of lineage committed BFU-E (Burst Forming Unit-Erythrocyte) and CFU-GM (granulocyte, macrophage) (Fig. 1F). The reduction in frequency of most immature CFU and the higher frequency of lineage positive cells suggest that exogenous Wnt3a protein promotes rather than inhibits growth factor-driven differentiation of CD34^+^ cells in expansion cultures. Next, we evaluated the dose-response relationship of Wnt signaling on the in vitro expansion of CD34^+^ cells. At the lowest concentration of 25 ng/ml, Wnt3a had no effect on the expansion of CD34^+^ cells relative to control SFT cultures (Fig. 1G). On the other hand, the highest concentration of 2500 ng/ml Wnt3a protein resulted in a decline of total cell number (not shown) and a complete loss of CD34^+^ cells (Fig. 1G). The effect of Wnt3a on the repopulating ability of the expanded CD34^+^ cells was assessed by transplantation of the expanded population of cells into sublethally irradiated NSG mice after 7 days of culture. All transplanted mice showed engraftment (defined as >0.1% human CD45^+^ cells in the peripheral blood at 7 weeks after transplantation). However, the kinetics of human chimerism development in peripheral blood appeared delayed when cells were cultured in the presence of Wnt3a (Fig. 1H). Lower levels of human chimerism were also observed in the bone marrow of mice 17 weeks after transplantation of cells cultured in the presence of Wnt3a (Fig. 1I, 36.9% versus 10.1% respectively, p<0.05). This indicates that Wnt3a protein reduces the long-term repopulation ability of cultured CD34^+^ cells.

### Wnt3a reduces growth factor-driven expansion of HSC

**Fig 2:**
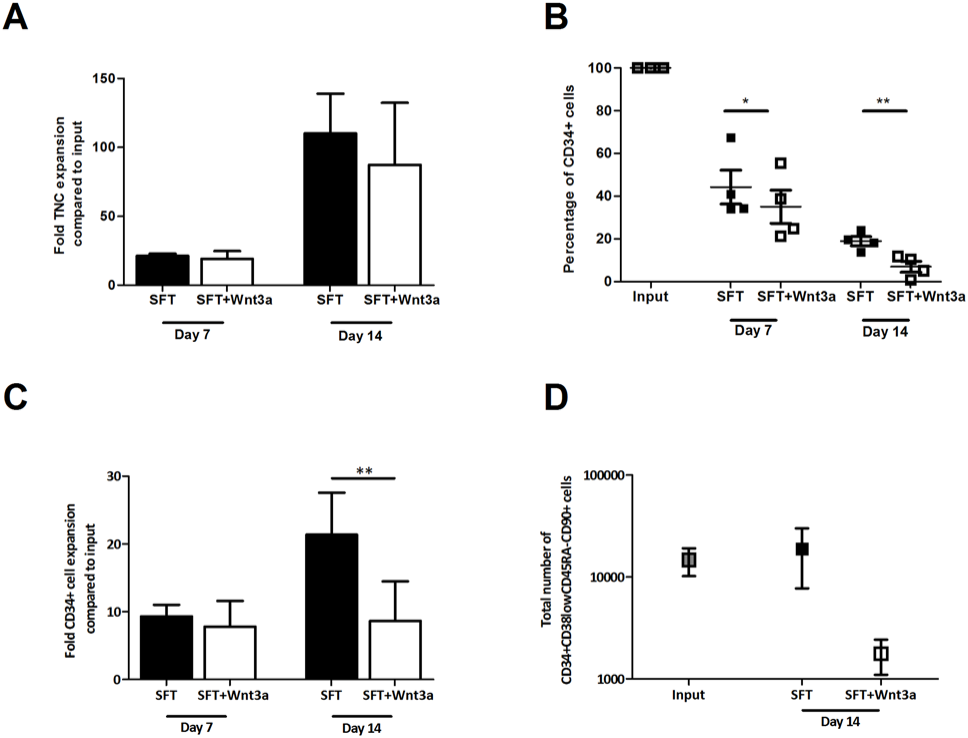
Wnt3a reduces growth factor-driven expansion of HSC. UCB-derived DAPI^-^Lin^-^CD34^+^CD38^low^CD45RA^low^CD90^+^-cells were sorted out of CD34-selected cells and were cultured in SFT medium with or without Wnt3a. Flowcytometric analysis was performed at day 7 and 14. Depicted are (A) the total nucleated cell expansion in SFT and SFT+Wnt3a medium at 7 and 14 days of culture (n = 4), (B) the CD34^+^ cell frequency during culture (n = 4), (C) the fold expansion of CD34^+^ cells at day 7 and 14 of culture (n = 4) and (D) the total number of DAPI^-^Lin^-^CD34^+^CD38^low^CD45RA^low^CD90^+^-cellsat input and after 14 days of culture in SFT or SFT+Wnt3a medium (n = 4). * p<0.*05*, ** p<0.*01*

CD34^+^ cells constitute a heterogeneous population, including only a minor fraction of the most immature HSC subset. Wnt3a may act differentially on primitive HSC and CD34^+^ cells with committed progenitor properties A putative differentiation-inhibiting effect of Wnt3a on HSC may be obscured by a differentiation-inducing effect on the large population of committed progenitor cells in culture. To study the effects of Wnt3a on the most immature HSC subset, we expanded Lin^-^CD34^+^CD38^low^ CD45RA^low^CD90^+^-cells, highly enriched for HSC[30] in the presence or absence of Wnt3a. A similar effect of Wnt3a was observed on the expansion of the sorted Lin^-^ CD34^+^ CD38^low^ CD45RA^low^ CD90^+^-cells. A robust total nucleated cell expansion, approximately 100-fold, was observed regardless of the presence of Wnt3a (Fig. 2A). However, Wnt3a again accelerated the decline in the frequency of CD34^+^ cells (Fig. 2B, p<0.05 and p<0.01 for 7 and 14 days of culture, respectively) and led to a significantly reduced expansion of CD34^+^ cells (Fig. 2C, p<0.01). Moreover, Wnt3a strongly reduced the number of Lin^-^CD34^+^ CD38^low^ CD45RA^low^ CD90^+^-cellsobtained after culture, while these cells were maintained in the absence of Wnt3a (Fig. 2D). These data suggest that Wnt3a inhibits the expansion of both multilineage committed progenitors and HSC.

### Prolongation of Wnt3a activity does not result in increased expansion of CD34+ cells

**Fig 3:**
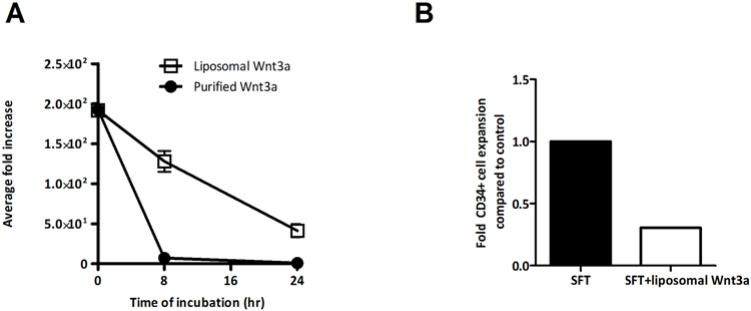
Liposomal Wnt3a reduces expansion of CD34^+^ cells. (A) Purified Wnt3a and liposomal Wnt3a were incubated for 0, 8, and 24 hours at 37°C in cell culture media, and transferred to LSL cells. Remaining Wnt activity was assayed by luminescence measurements. Activity plot displays average increase of luminescence over incubation time relative to background (n = 10). (B) CD34^+^ cell expansion in SFT medium with or without liposomal Wnt3a after 7 days of culture (n = 1).

Purified Wnt3a has been shown to have a half-life that is considerably shorter than 24 hours upon dilution in serum free media[32]. Thus, daily addition of Wnt3a protein to cell cultures would result in intermittent rather than continuous activation of the pathway. A possible explanation for our observations of reduced HSPC expansion in response to daily Wnt3a addition is that these intermittent pulses are unable to inhibit HSC differentiation. At the same time, Wnt signals may promote the differentiation of more mature CD34^+^ cells, leading to an overall reduction of HSPC. The stability of Wnt3a protein can be increased by association with liposomes[33, 34], and we therefore tested whether such stabilized Wnt ligands were able to prevent the decline in HSPC that we observed in response to regular Wnt3a protein. We compared the stability of purified Wnt3a protein and of Wnt3a protein associated with liposomes by incubating the proteins in serum free medium at 37°C and assessing the remaining Wnt-activity at several time points by a luciferase reporter assay. Whereas purified Wnt3a lost its activity within 8 hours, liposomal Wnt3a retained significant activity after 24 hours (Fig. 3A). Despite this increased stability however, liposomal Wnt3a induced a 3.3-fold decline of CD34^+^ cell expansion (Fig. 3B), similar to purified Wnt3a. In conclusion, prolongation of Wnt3a activity does not result in increased expansion of CD34^+^ cells.

### Enhanced differentiation of CD34^+^ cells is dependent on activation of the canonical Wnt pathway

**Fig 4:**
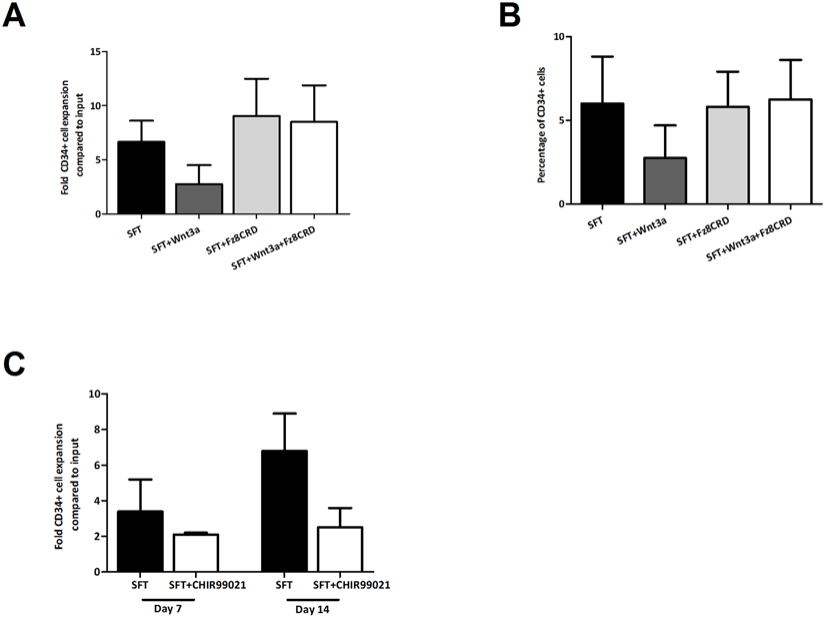
Wnt3a-mediated inhibition of HSPC expansion is due to activation of the canonical Wnt3a pathway. (A) Expansion of CD34^+^ cells after 7 and 14 days of culture in SFT, SFT+Wnt3a, SFT+Fr8CRD and SFT+Wnt3a+Fr8CRD (n = 2). (B) CD34^+^ cell frequency within the TNC population during these cultures (n = 2). (C) CD34^+^ cell expansion in SFT and SFT+CHIR99021 after 7 and 14 days of culture (n = 2).

Binding of Wnt3a to its receptor can be blocked with the Wnt antagonist Fz8CRD, a soluble domain of the Wnt receptor that sequesters Wnt proteins[31]. To demonstrate that the observed effects of Wnt3a were indeed dependent on binding to its receptor on HSPC, CD34^+^ cells were cultured with or without Wnt3a and/or Fz8CRD. The negative effect of exogenous Wnt3a on the expansion of CD34^+^ cells and the decline in frequency of CD34^+^ cells during culture was reversed by the addition of Fz8CRD. (Fig. 4A and 4B). An alternative way to activate the canonical Wnt pathway is by inhibiting glycogen synthase kinase 3β (GSK3β) which results in stabilization of the cytoplasmic β-catenin pool and subsequent transfer to the nucleus. To confirm that activation of the canonical Wnt pathway by exogenous Wnt3a underlies the inhibitory effects of Wnt3a on the expansion of CD34^+^ cells we used a synthetic GSK3β-inhibitor (CHIR99021). Addition of CHIR99021 supressed the expansion of CD34^+^ cells (Fig. 4C) to a similar extend as purified Wnt3a protein (Fig. 1D). Collectively these data show that the reduced expansion of CD34^+^ cells is due to binding of Wnt3a protein to its receptor on HSPC and subsequent activation of canonical Wnt pathway.

### Wnt3a inhibits expansion of CD34+ cells driven by the Aryl hydrocarbon receptor antagonist Stemregenin1

**Fig 5:**
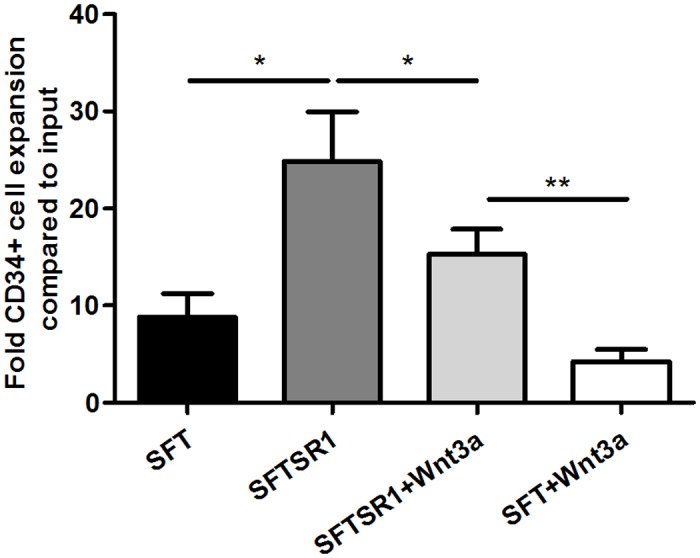
Wnt3a inhibits SR1-enhanced CD34^+^ cell expansion. UCB-derived CD34^+^ HSPC were cultured for 14 days in SFT or SFTSR1 medium with or without the addition of Wnt3a. Flowcytometric analysis was performed at day 14. Depicted is the CD34^+^ cell expansion after 14 days of culture (n = 5). * p<0.*05*, ** p<0.*01*

The Aryl hydrocarbon Receptor (AhR) is implicated to play a role in the proliferation and differentiation of HSPC. AhR-KO mice have increased number of LSK cells in the bone marrow and these LSK are hyperproliferative[35] and hematopoietic progenitors of donor mice treated with the AhR agonist TCDD show impaired competitive engraftment[36]. In addition, the AhR antagonist StemRegenin1 (SR1) has been shown to effectively enhance the expansion of human HSPC[37, 38]. We included SR1 in our cultures as a positive control for enhancing growth factor-driven CD34+ cell expansion and in addition, to evaluate putative cooperative effects of the Wnt and AhR signalling pathways. Expectedly, SR1 indeed promoted CD34^+^ cell expansion to a similar extent as reported before[37], indicating the suitability of our SFT medium, while Wnt3a reduced CD34^+^ cell expansion (Fig. 5). Addition of both Wnt3a and SR1 resulted in an increased expansion compared to Wnt alone, but a reduced expansion compared to SR1 alone (Fig. 5, p<0.01 and p<0.05 respectively), showing that even in the presence of AhR pathway inhibition, Wnt3a suppresses the expansion of CD34^+^ cells.

## Discussion

The effects of Wnt signaling on human HSPC proliferation, differentiation and survival are still poorly understood. In this study, we show that Wnt3a protein suppresses rather than promotes growth factor-driven expansion of UCB-derived CD34^+^ cells in serum-free expansion cultures. Wnt3a induced accelerated differentiation of both HSC and multipotent progenitors, resulting in the production of more differentiated cells. The reduced expansion of human HSPC, appeared to be dependent on activation of the canonical Wnt signaling pathway upon binding of Wnt3a to its receptor at the cell surface.

Our findings compare well to those by Nemeth et al., who observed a decrease in expansion of mouse LSKI (Lineage negative, Sca-1^+^, c-kit^+^, IL-7Rα^-^) cells after culture in serum-free medium containing SCF, Flt3L and Wnt3a compared to culture conditions with growth factors only. In addition, they showed no enhanced repopulation capacity of Wnt3a-cultured LSKI cells compared to control-cultured cells[25]. Earlier, several studies showed that overexpression of beta-catenin in HSC resulted in a functional defect of hematopoiesis and loss of repopulating activity[27, 39]. These results are in line with our observations of enhanced differentiation of HSPC upon stimulation of the canonical Wnt pathway. However, Malhotra et al. reported that overexpression of activated beta-catenin may expand the pool of HSC, both phenotypically and functionally in long term cultures[40]. In addition, overexpression of the Wnt inhibitor Dickkopf-related protein 1 (Dkk1) was shown to significantly impair the self-renewal capacity of adult HSC[21, 22]. How to reconcile these contradictory results? Although at first sight contradictory, the many differences in types of cultures, cells growth factors added may explain some of the observed differences.

Willert et al. and Reya et al. earlier showed that purified Wnt3a induced proliferation, while inhibiting differentiation of HSC in growth-factor based cultures of mouse LSKT (Lineage negative, Sca-1^+^, c-kit^+^, Thy-1.1^lo^)[24, 26]. However, they used cells derived from BCL2 transgenic mice. The BCL2 anti-apoptotic signal in HSPC may counteract possible apoptotic signals induced by Wnt3a signaling. Moreover, they used serum, which may supply additional signals that allow canonical Wnt signaling to exert a differentiation inhibitory effect or a more pronounced effect on self-renewal. It would compare well to several other studies[28, 41], which show that the presence of additional factors is required to balance the activated Wnt pathway. Trowbridge et al. showed enhanced long-term repopulation after treating mice with a GSK3beta inhibitor, which regulates the canonical Wnt pathway and several other pathways as well[41]. Perry et al. showed that the combination of a PTEN deletion and activation of beta-catenin results in enhanced self-renewal and expansion of HSC[28]. Another important pathway involved in HSC self-renewal and inhibition of differentiation is the pathway initiated by the aryl hydrocarbon Receptor (AhR). It is now well established that AhR-KO mice have increased number of LSK cells in the bone marrow and these LSK are hyperproliferative[35], while hematopoietic progenitors of donor mice treated with AhR agonist TCDD show impaired competitive engraftment[36]. In addition, the AhR antagonist StemRegenin1 (SR1) promotes expansion of human hematopoietic stem cells[37, 38]. We evaluated whether combined inhibition of the AhR pathway and activation of the Wnt pathway would result in enhanced expansion of HSPC. However, Wnt signaling also reduced the expansion of CD34^+^ HSPC in the presence of the aryl hydrocarbon receptor antagonist SR1. Another important factor that may affect the response of HSPC to Wnt signaling is the level of oxygenation. Expansion cultures are usually performed at normoxic levels. However, it is well established that the response of HSPC to hematopoietic cytokines is different under normoxic and hypoxic conditions, which mimics the in situ bone marrow environment[42]. The effect of stimulation of the canonical Wnt pathway on HSC expansion may be modulated by the level of oxygenation, which was already shown for the effect of the Wnt4-dependent pathway on the functional capacities of mesenchymal stem cells[43].

Apart from other cytokines and pathways involved, the dose and timing of Wnt activation might play an important role. Our data show that both intermittent pulses of Wnt3a and more continuous exposure to Wnt3a have a similar negative effect on the expansion of CD34^+^ cells in serum-free expansion cultures. Luis et al reported that Wnts are tightly regulated in a dose-dependent fashion[44], which may affect their biological activity. Thereby, varying results (exerted via the canonical Wnt pathway) may result from different levels of Wnt signaling, such as can be achieved in different experimental conditions. The optimal level of Wnt signaling for HSC was suggested to be only slightly increased over normal physiological values, while higher levels resulted in impaired engraftment potential of HSC fashion[44].

Collectively, we show that exogenous Wnt3a proteins reduces the expansion of human HSPC in serum-free growth factor-driven HSPC expansion cultures by promoting their differentiation without apparently affecting their proliferation or survival. It cannot be excluded that additional signals, such as the induction of a hypoxic cellular response, are needed for a possible positive effect of canonical Wnt signaling on HSPC expansion. Future studies may address the fragile balance between canonical Wnt signaling and other pathways to determine whether combined activation of Wnt signaling and other signaling pathways may promote human HSPC expansion.
